# Foam-Porous Alginate-Bentonite Beads Coated with Gamma-Irradiated Chitosan for Selective Chlorophyll Removal with Preservation of Plant Bioactives

**DOI:** 10.1021/acsomega.5c11997

**Published:** 2026-03-04

**Authors:** Titiya Meechai, Pitchapa Pittayavinai, Narudom Srisawang, Jintapat Nateewattana, Tanutta Amnuaywattanakul, Phitchan Sricharoen

**Affiliations:** 1 Faculty of Dentistry, 232369 Bangkokthonburi University, Thawi Watthana, Bangkok 10170, Thailand; 2 Division of Health, Cosmetic and Anti-Aging Technology, Faculty of Science and Technology, 187381 Rajamangala University of Technology Phra Nakhon, Bangkok 10800, Thailand

## Abstract

Excess chlorophyll in plant ethanol extracts can compromise analytical accuracy and restrict industrial applications in food, herbal, and cosmetic formulations. This study developed foam-porous alginate-bentonite beads coated with gamma-irradiated chitosan (FP-Alg/Bent-gCS) as a selective, reusable biocomposite adsorbent for chlorophyll removal while preserving plant bioactives. The beads were created using CO_2_ foaming, cross-linked with CaCl_2_, reinforced with bentonite, and coated with gamma-irradiated chitosan (0.5%, pH 5.5). The resulting beads demonstrated a high surface area (75.6 m^2^·g^–1^) and pore volume (0.096 cm^3^·g^–1^), as confirmed by BET analysis. FTIR spectra indicated the presence of hydrogen bonding and electrostatic interactions among hydroxyl, carboxyl, and amino groups. Additionally, XRD and HRTEM confirmed the formation of a semicrystalline foam-like structure. When applied to kale extract in 50% ethanol (25 mL), the beads achieved an impressive 88.4% total chlorophyll removal in just 30 min at a dosage of 5 g. This process followed pseudo-second-order kinetics, suggesting chemisorption via electrostatic attraction between −NH_3_^+^ groups and chlorophyll molecules. Colorimetric assays for total phenolic content (TPC) and total flavonoid content (TFC) revealed no significant reduction in polyphenols or flavonoids, confirming that the removal of pigments was selective and did not lead to loss of bioactives. The beads maintained over 85% removal efficiency through five reuse cycles, with only minor surface deformation observed after the sixth cycle, consistent with XRD and EDS analyses that showed structural stability. In conclusion, the FP-Alg/Bent-gCS beads represent a low-cost, environmentally friendly, and sustainable method for selectively removing chlorophyll while preserving bioactives. They present a promising material for green extraction and purification processes in the food, herbal, and cosmetic industries.

## Graphical abstract

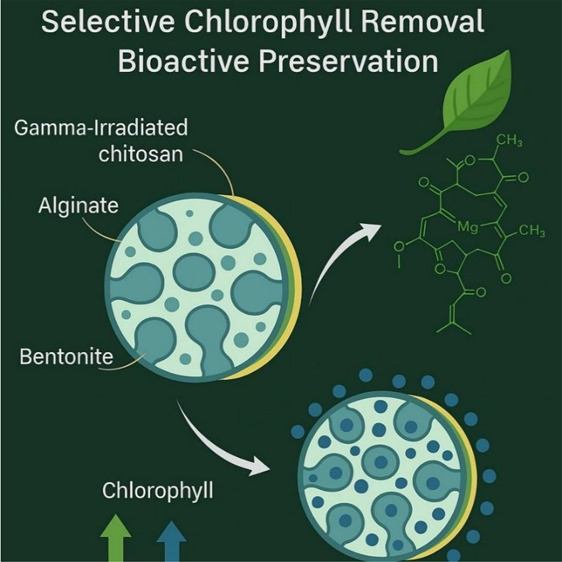

## 1 Introduction

Chlorophyll, a magnesium-containing tetrapyrrole, is the primary pigment found in leafy plants and herbal extracts.^1–3^ While it plays a crucial role in photosynthesis, its persistence in ethanol extracts presents significant challenges for industrial applications and analytical workflows. High levels of chlorophyll lead to undesirable dark green coloration, accelerate photo-oxidative degradation, and interfere with chromatographic analyses (such as HPLC and LC-MS/MS) by overlapping with bioactive signals. Therefore, dechlorophyllization is a critical step in producing high-quality extracts for food, cosmetic, and nutraceutical products.^4^

Conventional methods, including activated carbon, silica gel, bentonite slurries (also known as bleaching earth) and hydroxyapatite can effectively reduce dyes and pigments but often lack selectivity.^5^ These methods often remove valuable bioactive compounds, such as polyphenols, flavonoids, carotenoids, as well as chlorophyll. Furthermore, these powder-based adsorbents are challenging to separate, nonreusable, and produce significant waste, such as the spent bleaching earth generated in edible oil refining.^6,7^

More advanced techniques, including solid-phase extraction (SPE) and centrifugal partition chromatography (CPC), have shown higher efficiency in pigment removal.^8,9^ However, these methods require large solvent volumes, are costly, and necessitate specialized equipment, rendering them impractical for large-scale industrial use. In our previous research, we investigated composite beads composed of alginate, mesoporous silica (SBA-15), and chitosan for the adsorption of dyes and pollutants.^10–12^ These findings highlighted that bead systems are highly tunable platforms capable of structural and chemical modifications to selectively capture target molecules. However, native chitosan has limited solubility and long polymer chains, which can lead to uneven coatings and slow diffusion. In contrast, gamma-irradiated chitosan offers distinct advantages: radiation treatment reduces its molecular weight, enhances its solubility in mild acids, and increases the density of protonated amine groups, enabling the formation of thin, uniform coatings and improving electrostatic interactions.^13^ As a result, gamma-irradiated chitosan, a natural biopolymer composed of β-1,4-linked 2-acetamido-d-glucose and β-1,4-linked 2-amino-d-glucose,^14^ has been shown to enhance the selective adsorption of hydrophobic pigments, such as chlorophyll.^15,16^

To date, no studies have combined foam-templated porous beads with bentonite reinforcement and gamma-irradiated chitosan coating into a single composite system for the selective dechlorination of pollutants. This study aims to develop foam-porous alginate-bentonite beads fabricated using CO_2_ foaming, which will be coated with irradiated chitosan. This composite system will serve as a selective gate for chlorophyll removal while preserving bioactive compounds.

## 2 Materials and Methods

### 2.1 Materials

Sodium alginate (medium viscosity), sodium bicarbonate (NaHCO_3_, 99%), calcium chloride (CaCl_2_, anhydrous), glacial acetic acid, and ethanol (95%) were obtained from Merck (Germany). Sodium-type bentonite clay was sourced from Sigma-Aldrich (USA). Chitosan was irradiated with γ rays at a sterilizing dose of 40 kGy, resulting in a molecular weight of 190 kDa and a degree of deacetylation of 95%. This chitosan was provided by the Thailand Institute of Nuclear Technology (Public Organization). All reagents were of analytical grade and were used without further purification.

### 2.2 Preparation of Foam-Porous Alginate-Bentonite Beads (FP-Alg/Bent-gCS Beads)

Foam-porous beads were prepared using a CO_2_ foaming strategy. A 2% w/v sodium alginate solution (100 mL) was prepared by dissolving alginate powder in distilled water with magnetic stirring at room temperature. Bentonite (0.30 g per 100 mL of the alginate solution) was predispersed in 5 mL of deionized (DI) water to form a slurry, which was then added to the alginate solution. Sodium bicarbonate (0.70 g/100 mL) was included as a gas-forming agent. The resulting suspension was extruded dropwise through a syringe pump (using a 22G needle) into a gelling bath containing 2% w/v calcium chloride (CaCl_2_) and 0.3% v/v acetic acid, while stirring gently. The instantaneous release of CO_2_ during cross-linking created macroporosity within the hydrogel beads. The beads were allowed to harden for 20 to 30 min, then rinsed with deionized water to remove excess calcium ions (Ca^2+^) and any residual reagents.

### 2.3 Surface Coating with Gamma-Irradiated Chitosan

The beads were coated with gamma-irradiated chitosan to create a selective outer gate layer. The preparation and coating procedure is illustrated in Figure 1. A 0.5% w/v solution of gamma-irradiated chitosan was prepared using 0.5% v/v acetic acid, and the pH was adjusted to 5.4–5.8 with 0.1 M NaOH. The preformed beads were immersed in the chitosan solution for 5–10 min, gently agitating during immersion. Afterward, the beads were rinsed briefly with deionized water to remove any unbound chitosan. The coated beads were then stored at 4 °C until further use.

**Figure 1:**
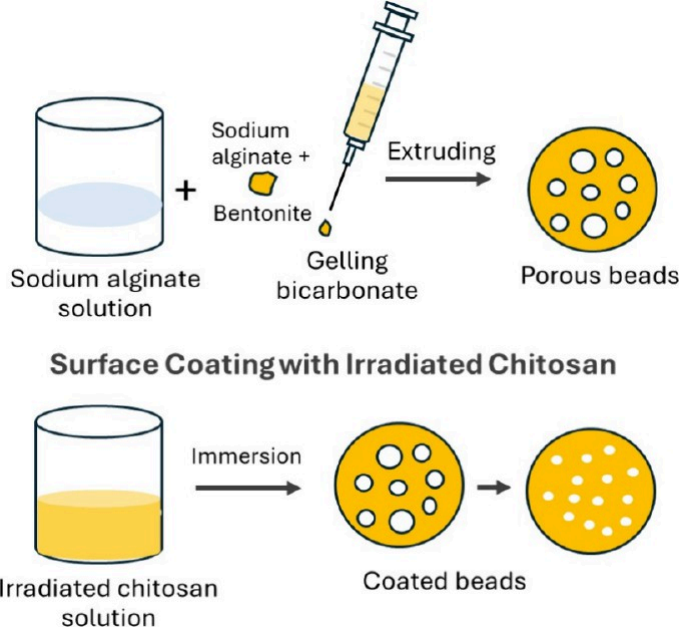
Schematic illustration of the preparation and coating of foam-porous alginate-bentonite beads with gamma-irradiated chitosan. Created by Titiya Meechai.

### 2.4 Chlorophyll Removal Assay

Model plant extracts were prepared by homogenizing fresh kale leaves in 95% ethanol, then diluting to 50% v/v. Beads were added to 25 mL of the extract, and the mixture was incubated for 10–30 min under gentle stirring. After incubation, the beads were separated by gravity filtration using Whatman No.1 qualitative filter paper, and the filtrate was collected for chlorophyll analysis. The chlorophyll content was determined spectrophotometrically using a DLAB SP-UV1000 Spectrophotometer by measuring the absorbance at 663 and 645 nm (Arnon-type chlorophyll equations).^17–19^ The concentrations of chlorophyll a, chlorophyll b, and total chlorophyll (mg/L) were calculated using the relevant equations:

$$\mathrm{Chl}\;a=12.25\times A_{663}-2.79\times A_{645}$$

$$\mathrm{Chl}\;b=21.50\times A_{645}-5.10\times A_{663}$$

totalchlorophyll=Chla+Chlb

Removal efficiency (%) was calculated as

$$\mathrm{removal}\;\mathrm{efficiency}\left(\%\right)=\frac{\left(C_{0}-C_{e}\right)}{C_{0}}\times 100$$

Where *C*_0_ and *C*_t_ are the initial and final chlorophyll concentrations, respectively.

### 2.5 Bioactive Retention Analysis

The retention of bioactive compounds after chlorophyll removal was evaluated using the following methods:

#### Total Phenolic Content (TPC)

Determined using the Folin-Ciocalteu method with gallic acid as the standard. The results are expressed as milligrams of gallic acid equivalents per gram of extract (mg GAE/g extract).^20,21^

#### Total Flavonoid Content (TFC)

This was measured using the aluminum chloride colorimetric method, with quercetin as the standard. The results are presented as milligrams of quercetin equivalents per gram of extract (mg QE/g extract).^22^

The retention percentage is calculated by dividing the concentration after treatment by the initial concentration and expressing the result as a percentage.

### 2.6 Reusability of Beads

After each adsorption cycle, the used beads were regenerated by soaking them in 50% ethanol for 10 min, rinsed twice with deionized water, and reused in subsequent adsorption cycles. The efficiency of chlorophyll removal and the retention of bioactive compounds were measured.

### 2.7 Structural and Physicochemical Characterization

High-resolution transmission electron microscopy (HRTEM) and scanning transmission electron microscopy (STEM) analyses were performed using a JEOL JEM-ARM200F microscope in HRTEM and STEM modes, respectively. HRTEM was used to examine the nanoscale morphology and internal structure of composite beads, focusing on the dispersion of bentonite platelets within the alginate-chitosan matrix. STEM was performed using backscattered electron imaging (BEI-STEM) and high-angle annular dark-field (HAADF-STEM) modes, which provided Z-contrast to distinguish clay-rich regions from the organic polymer matrix. Selected-area electron diffraction (SAED) was used to assess crystallinity, distinguishing amorphous polymer domains from crystalline silicate phases. Overall, these electron microscopy techniques yielded valuable morphological and structural insights, enhancing our understanding of clay dispersion and partial intercalation within the biopolymer network. Fourier-transform infrared spectroscopy (FTIR; Bruker Tensor 27) was used to investigate interactions between the functional groups of alginate, bentonite, and chitosan. X-ray diffraction (XRD) analysis was performed using a Bruker D8 Advance A25 diffractometer with a Ni filter and Lynxeye multistrip detector. The Brunauer–Emmett–Teller (BET) surface area was measured at 77.3 K with nitrogen (N_2_) using a Quantachrome Instruments v11.0 system. Lastly, the swelling behavior of the beads was assessed in a 50% ethanol solution at 25 °C.

## 3 Results and Discussion

### 3.1 Structural and Physicochemical Characterization

Figure 2a illustrates the overall appearance of the prepared beads, which were spherical, translucent, and uniform in size, measuring approximately 2–3 mm in diameter. The smooth surface indicates efficient gelation during Ca^2^-mediated cross-linking. Figure 2b presents a magnified image of a single bead’s surface, revealing microbubbles and micropores within the matrix. These pores were formed by an in situ foaming reaction between sodium bicarbonate and acetic acid during gelation, yielding a lightweight, highly porous texture. Figure 2c shows the internal cross-section of a bisected bead, confirming the existence of interconnected pores throughout the structure. The uniform distribution of pores demonstrates that CO_2_ generation was consistent across the droplet, forming a sponge-like internal morphology that is favorable for the rapid diffusion and adsorption of chlorophyll molecules. The maintenance of intact spherical shapes even after cutting indicates that the hydrogel network has good mechanical stability. Similar porous patterns have been observed in CO_2_-foamed alginate systems, where gas generation serves as a physical templating mechanism. The preserved transparency and structure suggest that the reinforcement from bentonite and the gamma-chitosan coating have enhanced cross-linking density and prevented structural collapse.^23,24^

**Figure 2:**
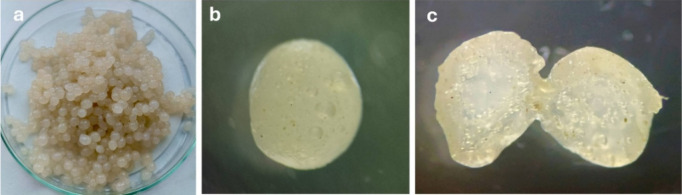
Optical and microscopic morphology of FP-Alg/Bent-gCS beads: (a) surface appearance of uniform spherical beads; (b) surface microstructure showing CO_2_-formed micropores; (c) internal cross-section confirming interconnected porous channels. Photograph courtesy of Titiya Meechai. Copyright 2025.

The multiscale electron microscopy and EDS mapping clearly confirm the presence of a well-integrated clay–polymer composite. HRTEM imaging reveals thin lamellar silicate platelets that are both exfoliated and partially intercalated within the alginate-chitosan matrix (Figure 3). Local lattice fringes indicate the presence of nanocrystalline domains, while the surrounding polymer remains amorphous mainly. STEM-BEI and HAADF images reveal bright Z-contrast in clay-rich areas, embedded within a darker organic matrix, indicating intimate interfacial contact without any macroscopic voids. The selected-area electron diffraction (SAED) pattern displays diffuse rings with fine spots, consistent with an amorphous polymer that embeds polycrystalline silicate/Ca-alginate microdomains. Elemental mapping reveals a homogeneous distribution of silicon and aluminum (from bentonite) in the bright regions, along with calcium present both within and along the matrix. This reflects the cross-linking of alginate (in an “egg-box” structure) with Ca^2+^ ions, with partial anchoring to clay surfaces. This exfoliated and partially intercalated architecture explains the rapid adsorption kinetics and high chlorophyll removal capacity observed, as it provides abundant accessible sites and continuous diffusion pathways while maintaining mechanical integrity during cyclic use.

**Figure 3:**
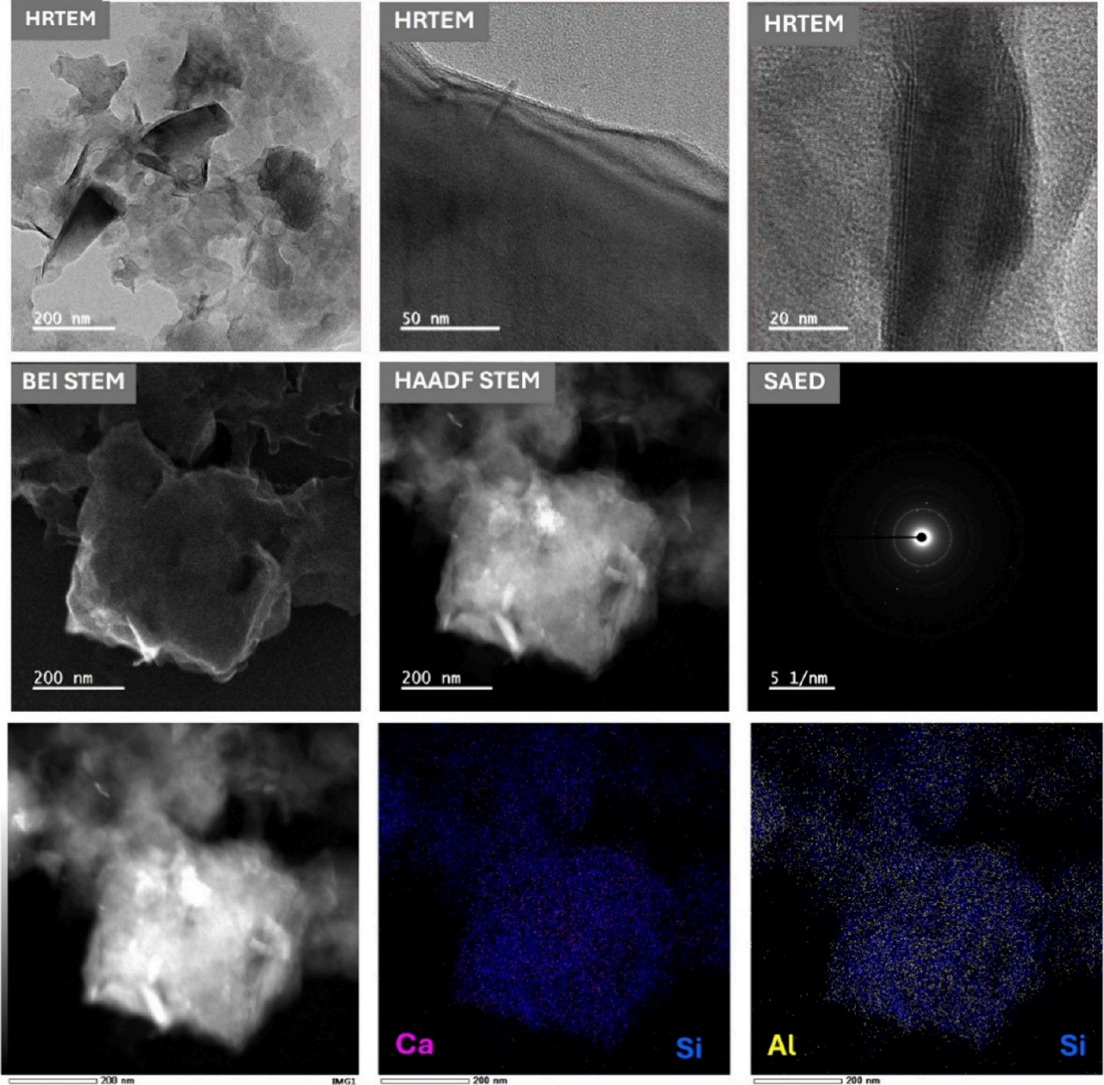
HRTEM, BEI-STEM, HAADF-STEM, SAED, and EDS mapping of FP-Alg/Bent-gCS beads.

The upper Selected Area Electron Diffraction (SAED) pattern exhibits diffuse rings with faint spots (Figure 4), indicating a hybrid structure composed of amorphous and polycrystalline domains, resulting from the coexistence of bentonite and biopolymer domains. The lower SAED pattern exhibits sharper spots, indicating localized nanocrystalline domains, which may originate from bentonite silicate layers or calcium alginate microcrystals formed during cross-linking. This combination of amorphous and crystalline regions creates a stable hybrid clay–polymer matrix, where the crystalline domains provide mechanical reinforcement and the amorphous phase facilitates the diffusion and adsorption of chlorophyll molecules.

**Figure 4:**
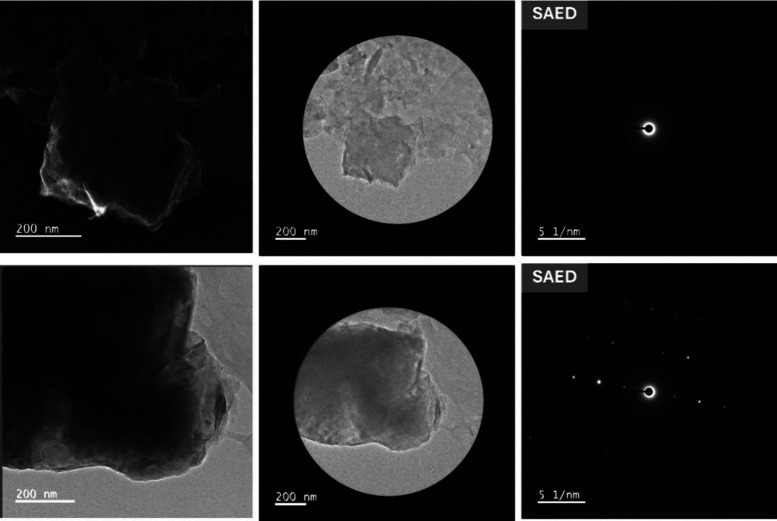
SAED analyses of FP-Alg/Bent-gCS beads showing localized crystallinity and ordered silicate lamellae within the alginate-chitosan matrix.

The spectrum of FP-Alg/Bent-gCS beads shows characteristic absorption bands indicative of specific functional groups (Figure 5). Notably, O–H and N–H stretching vibrations appear around 3400 cm^–1^, while C–H stretching is observed at 2920 cm^–1^. Additionally, asymmetric and symmetric COO^–^ stretching bands are observed at 1625 cm^–1^ and 1410 cm^–1^, respectively, confirming interactions between alginate and chitosan. The band around 1030 cm^–1^ is attributed to the Si–O–Si stretching mode of bentonite.^25–27^ Furthermore, the reduced intensity of the −COO^–^ and −NH_2_ bands after coating suggests an electrostatic interaction between alginate and gamma-irradiated chitosan.^28–30^ These results confirm the successful formation of a stable composite network of alginate, bentonite, and chitosan through hydrogen bonding and ionic cross-linking.

**Figure 5:**
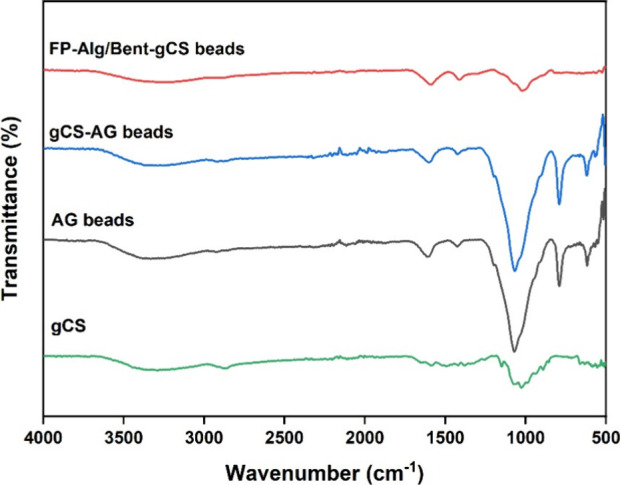
FTIR spectra of gCS, AG beads, gCS-AG beads, and FP-Alg/Bent-gCS beads.

The AG beads displayed distinct semicrystalline peaks at approximately 2θ = 21° and 35°, which correspond to the alginate polymer backbone (Figure 6). When coated with gamma-irradiated chitosan (gCS-AG beads), we observed a slight sharpening and shifting of the main peaks. Upon adding bentonite (FP-Alg/Bent-gCS beads), the prominent bentonite peaks at 6–7°, 19–21°, and 26–28° significantly broadened or diminished. This suggests partial exfoliation and intercalation of the silicate layers within the polymer matrix. These structural changes indicate the formation of an amorphous hybrid that enhances surface area and porosity. This is consistent with the results obtained from BET analysis and adsorption studies. Similar peak broadening and decreased crystallinity have been observed when bentonite is added to alginate- or chitosan-based matrices, as noted in previous studies on alginate-bentonite and chitosan-alginate composites. These structural changes are generally attributed to the partial exfoliation and intercalation of clay platelets within the polymer network, which disrupts the regular arrangement of silicate layers.

**Figure 6:**
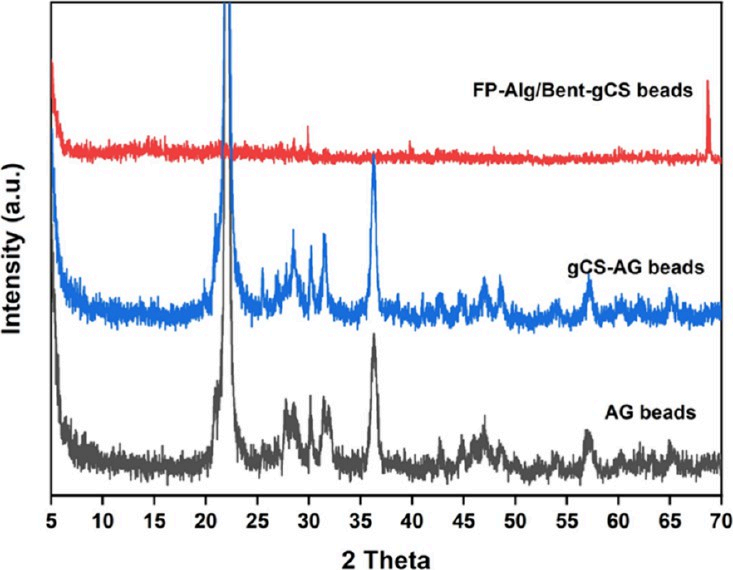
XRD patterns of AG beads, gCS-AG beads, and FP-Alg/Bent-gCS beads.

Nitrogen adsorption–desorption analysis reveals that all samples exhibit Type II isotherms, indicating structures ranging from nonporous to macroporous, characteristic of hydrogel-derived materials (Figure 7). The addition of bentonite and foam templating improved surface area and pore connectivity, as evidenced by increased nitrogen (N_2_) uptake and a narrower pore-size distribution in the FP-Alg/Bent-gCS beads. These results confirm the establishment of a foam-templated porous network that enhances adsorption performance. Table 1 showed that the FP-Alg/Bent-gCS beads had the highest specific surface area (75.614 m^2^·g^–1^) and pore volume (0.096 cm^3^·g^–1^) when compared to AG (alginate) and gCS-AG beads. The increased surface area and pore volume indicate that the CO_2_ foaming and bentonite reinforcement successfully created a mesoporous structure that is advantageous for chlorophyll adsorption. The increase in specific surface area and pore volume observed for the FP-Alg/Bent-gCS beads is consistent with previously reported alginate-bentonite and chitosan-based composite systems, in which clay reinforcement and polymer–clay interactions lead to a more open, accessible porous structure. Such enhancements in textural properties have been shown to correlate with improved adsorption performance.

**Figure 7:**
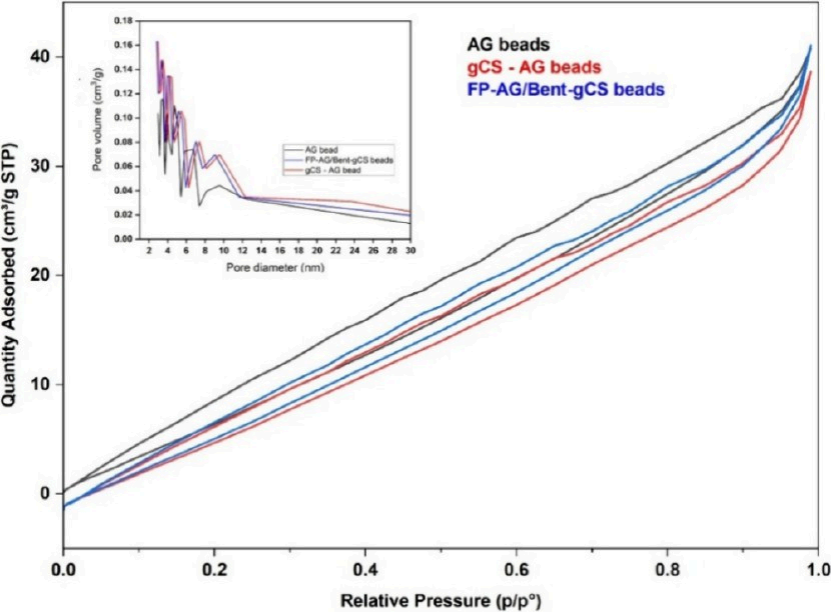
N_2_ adsorption–desorption isotherms and pore size distribution (inset) of AG beads, gCS-AG beads, and FP-Alg/Bent-gCS beads.

**Table 1:** BET Surface Area and Pore Structure of FP-Alg/Bent-gCS Beads

samples	surface area^a^, S_BET_ (m^2^·g^–1^)	pore volume^b^, Vp (cm^3^·g^–1^)	average pore diameter^b^, Dp (nm)
AG bead	51.447	0.058	2.969
gCS-AG bead	68.045	0.083	2.971
FP-Alg/Bent-gCS beads	75.614	0.096	3.021

These observations collectively indicate that the structural modifications from bentonite incorporation and chitosan coating enhance the adsorption capacity observed in this study.

### 3.2 Chlorophyll Removal Efficiency

The left image displays the plant extract solution before and after treatment with FP-Alg/Bent-gCS beads (Figure 8). Initially, the solution appears green, but the chlorophyll intensity decreases noticeably after treatment. The right image shows the beads after they have adsorbed the chlorophyll; their surfaces are visibly green, indicating successful uptake of chlorophyll pigments from the ethanol extract. These results confirm that the foam-porous alginate-bentonite structure, combined with gamma-irradiated chitosan coating, effectively adsorbs chlorophyll molecules while maintaining the integrity of the beads.

**Figure 8:**
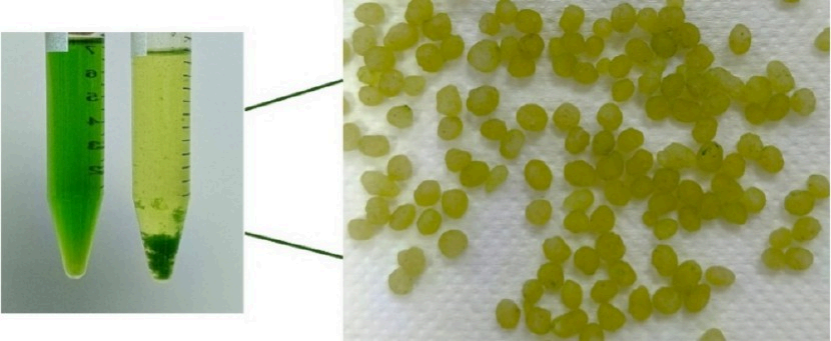
Chlorophyll adsorption behavior of FP-Alg/Bent-gCS beads. Photograph courtesy of Titiya Meechai. Copyright 2025.

The adsorption kinetics of chlorophyll from kale extract in a 50% ethanol solution showed a time-dependent decrease in total chlorophyll concentration. Increasing the bead dosage from 1.5 to 5.0 g significantly improved chlorophyll removal efficiency, reducing the total concentration from 20.09 mg/L to 2.33 mg/L within 30 min. The adsorption process occurred quickly during the first 10 to 20 min (Figure 8), followed by a gradual equilibrium phase. These results indicate that bentonite beads provide ample surface area and active adsorption sites for effective pigment removal, consistent with the Langmuir-type adsorption behavior observed in studies by Wang and Guo (2020).^31^

The results indicate that total chlorophyll concentration decreased significantly with both increased adsorption time and bead dosage (Figure 9). The chlorophyll removal efficiency reached 88.4% when 5.0 g of beads were used within 30 min, demonstrating the strong pigment-binding capacity of bentonite-based adsorbents. The adsorption process occurred rapidly during the first 15 to 20 min, likely due to abundant active sites and electrostatic attraction between the negatively charged chlorophyll molecules and the positively charged bead surfaces.^32^

**Figure 9:**
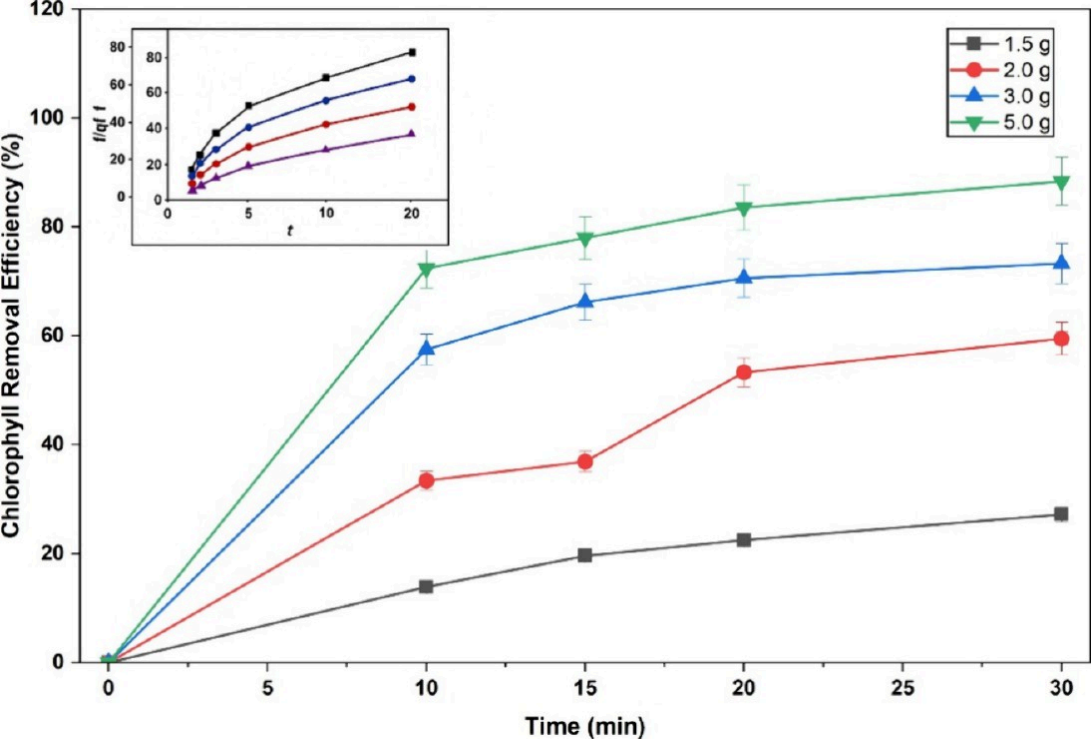
Chlorophyll removal (%) as a function of contact time for FP-Alg/Bent-gCS beads at different dosages (1.5–5.0 g) in 50% ethanol (25 °C; *V* = 25 mL). Inset: linear PSO plots (t/qt vs t) highlighting rapid uptake within 10–20 min and subsequent equilibrium.

For adsorption kinetics, the time-course of total chlorophyll revealed a rapid uptake within 10–20 min, followed by a gradual approach to equilibrium (30 min). Linearization of t/qt vs t indicated excellent agreement with the pseudo-second-order (PSO) model (Ho-McKay),^33^ yielding *q*_*e*_ = 0.10 mg.g^–1^ and *k*_2_ = 2.7 g.mg^–1^ min^–1^ for the 5.0 g dosage, and *q*_*e*_ = 0.14 mg.g^–1^, *k*_2_ = 1.48 g.mg^–1^ min^–1^ for 3.0 g. The PSO dominance suggests a site-limited interaction driven by electrostatic/complexation effects at the g-chitosan-coated surfaces and within the bentonite lamellae. In contrast, the pseudo-first-order (PFO) model showed poorer linearity than the PSO model, suggesting a less suitable description of the adsorption process. The corresponding PFO linear plots and fitting parameters are provided in the Supporting Information (Table S1 and Figure S1). These features are consistent with site-limited adsorption behavior, commonly described by Langmuir-type assumptions that assume the presence of a finite number of active sites, rather than formal Langmuir isotherm fitting. Similar interpretations have been reported for pigment adsorption in clay–polymer composite systems that exhibit rapid boundary-layer diffusion and pseudo-second-order kinetic behavior.^31,32,34^

Compared to conventional alginate beads, alginate-chitosan beads, and bentonite-based adsorbents described in the literature, FP-Alg/Bent-gCS beads exhibit superior efficiency in chlorophyll removal and faster adsorption kinetics. Traditional alginate beads exhibit limited affinity for hydrophobic pigments due to their predominantly hydrophilic carboxylate network, leading to lower chlorophyll uptake and slower diffusion rates. While alginate-chitosan systems enhance adsorption through electrostatic interactions, their dense polymer networks can restrict mass transfer. On the other hand, bentonite-based adsorbents demonstrate a high affinity for pigments due to their silicate layers, but they suffer from poor reusability and challenges with solid–liquid separation when used in powder form. The FP-Alg/Bent-gCS beads combine foam-induced macroporosity, clay reinforcement, and a positively charged gamma-irradiated chitosan coating. This combination provides a synergistic boost in surface accessibility, electrostatic attraction, and structural stability, allowing them to outperform previously reported systems.^25–27,31–33,35^

### 3.3 Retention of Bioactive Compounds

The retention of bioactive compounds was evaluated using the Folin-Ciocalteu and AlCl_3_ colorimetric methods to determine total phenolic content (TPC) and total flavonoid content (TFC), respectively (Figure 10). The TPC values before treatment were 62.5 ± 1.8 mg GAE/g extract, which decreased slightly to 59.2 ± 1.4 mg GAE/g after chlorophyll removal using F-Alg/Bent-gCS beads. Similarly, the TFC values changed from 41.7 ± 1.6 mg QE/g to 39.5 ± 1.3 mg QE/g, showing no significant difference (*p* > 0.05). These results indicate that hydrophilic polyphenolic and flavonoid compounds were largely preserved during the selective adsorption process. The gamma-irradiated chitosan layer provided electrostatic repulsion toward these polar bioactives while preferentially binding hydrophobic chlorophyll molecules through π–π and hydrophobic interactions. The foam-porous alginate-bentonite matrix further reduced steric hindrance and maintained molecular diffusion. This results in positively charged −NH_3_^+^ groups that preferentially bind to hydrophobic phytopigments, such as chlorophyll.^34,35^ Meanwhile, the hydrophilic bioactives are either electrostatically repelled or remain solvated in the ethanol–water medium. Furthermore, the foam-induced porosity of the alginate-bentonite matrix accelerates mass transfer and reduces the physical trapping of small hydrophilic molecules. The bentonite layers also stabilize the bead structure and modulate the surface charge distribution, minimizing nonspecific adsorption. These structural effects align with the report that porous hydrogel networks enhance the selective retention of phenolics during pigment purification processes.^26,36^ In summary, the FP-Alg/Bent-gCS beads function as a “selective adsorption gate”, effectively capturing chlorophyll while preserving the bioactivity of the extract. This capability is crucial for sustainable extraction and purification in the food and cosmetic industries.

**Figure 10:**
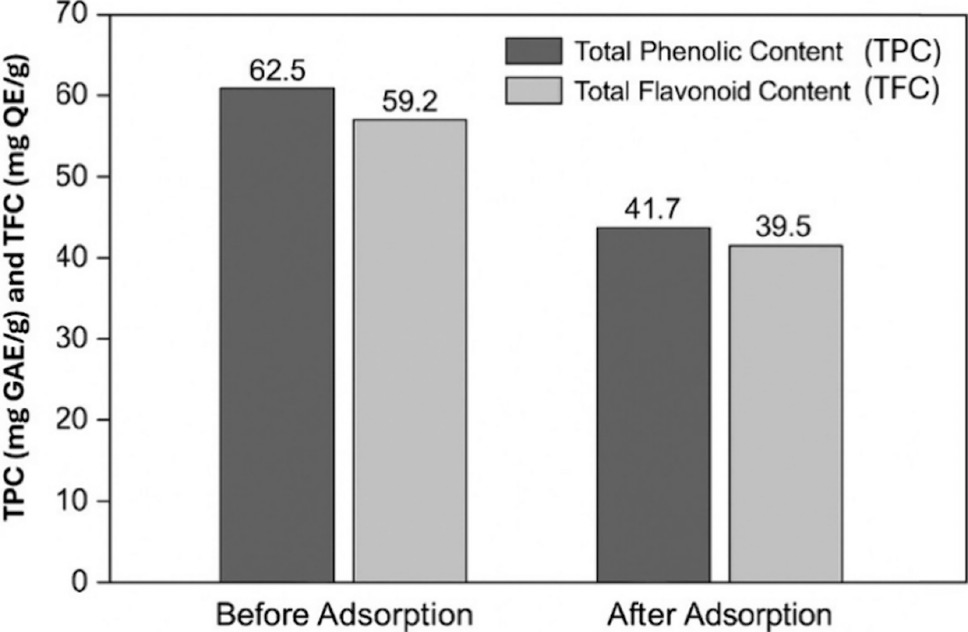
Total phenolic content (TPC) and total flavonoid content (TFC) before and after chlorophyll removal using FP-Alg/Bent-gCS beads.

### 3.4 Reusability

The reusability of the FP-Alg/Bent-gCS beads was assessed over seven consecutive adsorption–desorption cycles using 50% ethanol as the regenerating solvent. The beads demonstrated high chlorophyll removal performance throughout the first five cycles, showing no visible cracking or collapse. However, a gradual decline in performance became apparent during cycles six and seven, consistent with partial site saturation and minor surface changes resulting from repeated exposure to the solvent.

After five regeneration cycles (Figure 11), the chlorophyll removal efficiency of FP-Alg/Bent-gCS beads decreased from approximately 88% to 55%. This reduction in adsorption capacity can be attributed to partial saturation of binding sites and slight deformation of the bead matrix resulting from repeated exposure to ethanol. Visual observations confirmed that the beads retained their general shape but showed minor surface roughness and reduced mechanical integrity. This behavior is typical of alginate clay and chitosan-modified adsorbents regenerated with alcohols or mild eluents.^37^ To further support the observed durability, structural analyses were conducted after selected cycles. After adsorption, intensified absorption bands appear at approximately 3400 cm^–1^ (O–H/N-H stretching), 2920–2850 cm^–1^ (C–H stretching of phytol chains), and 1735–1650 cm^–1^ (C=O stretching from chlorophyll). This confirms the attachment of chlorophyll molecules through hydrogen bonding and electrostatic interactions with the beads. Key peaks for alginate (−COO^–^ at 1625 and 1410 cm^–1^), chitosan (amide II at 1550 cm^–1^), and bentonite (Si–O–Si at around 1030 cm^–1^) indicate the structural integrity of the beads. While the Mg–N vibration of chlorophyll is not detected in this region (below 500 cm^–1^), enhanced C=O and C–H peaks suggest pigment retention.

**Figure 11:**
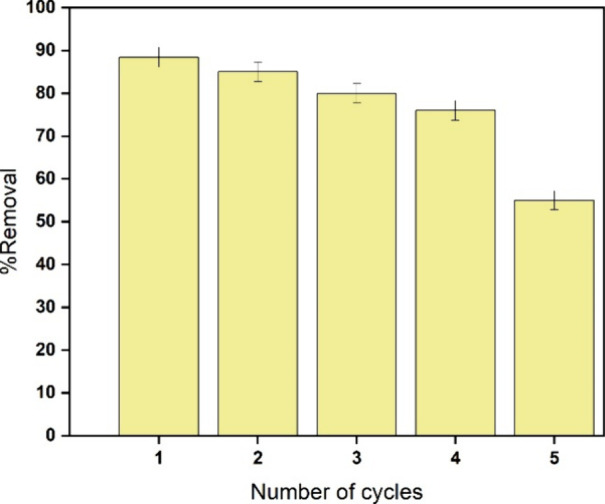
Reusability of FP-Alg/Bent-gCS beads over five adsorption–desorption cycles using 50% ethanol as the regenerating solvent. The chlorophyll removal efficiency decreased gradually from 88 to 55% after five cycles, corresponding to minor structural deformation and partial loss of active sites.

FTIR and XRD patterns indicate that the structural framework of the FP-Alg/Bent-gCS beads remained stable after adsorption, showing no significant degradation of the composite network. The HRTEM and HAADF-STEM images reveal layered and compact morphologies, suggesting that the lamellar integrity of bentonite within the polymer matrix has been maintained. Additionally, the EDS elemental maps confirm the presence of magnesium distributed throughout the structure, indicating successful adsorption of chlorophyll molecules that contain magnesium centers. (Figure 12). These findings align with prior reports on alginate/bentonite composites, in which the addition of bentonite layers enhances mechanical stability and modulates surface charge. This helps limit nonspecific adsorption and resist structural collapse during cycling.^26,38^

**Figure 12:**
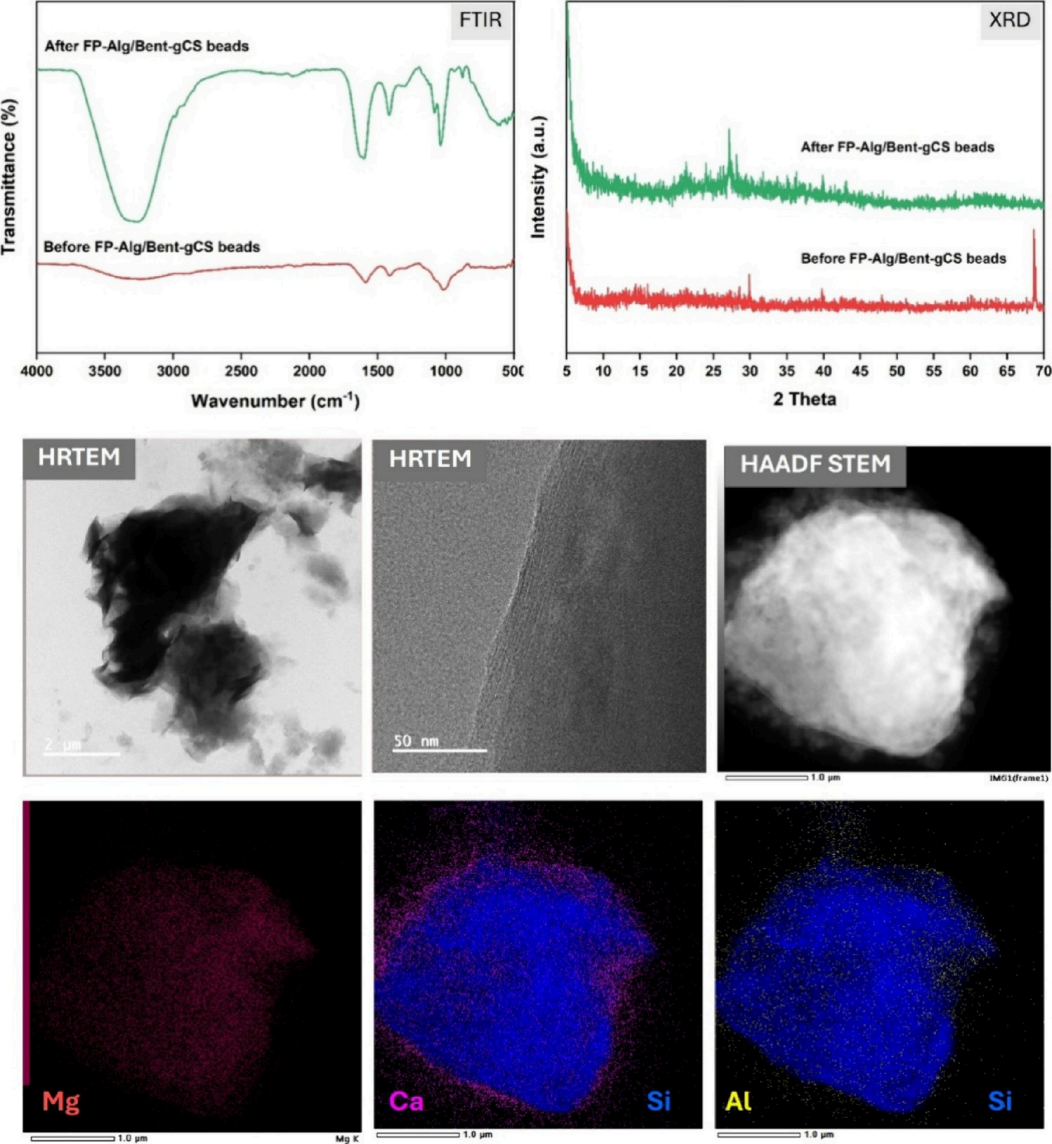
FTIR, XRD, and TEM–EDS analyses of FP-Alg/Bent-gCS beads after chlorophyll adsorption.

Mechanistically, the robust reusability can be attributed to three primary factors: (i) the Ca^2+^ mediated ionic gelation of alginate, which withstands multiple solvent exchanges, (ii) the reinforcement provided by bentonite, which stiffens the network and buffers swelling, and (iii) the gamma-irradiated chitosan coating, which preserves interfacial functionality in ethanol media. The existing literature on reusable biopolymer adsorbents supports this combination of stable cross-linking and gentle ethanol regeneration for pigment systems, including platforms for recyclable chlorophyll separation.^37^ Overall, FP-Alg/Bent-gCS beads can be efficiently reused for at least five cycles with only modest performance losses thereafter. They offer a practical, low-waste option for degreening plant extracts while maintaining structural integrity.^37^

### 3.5 Mechanistic Insights

The adsorption of chlorophyll onto the FP-Alg/Bent-gCS beads occurs through a synergistic mechanism that combines physical entrapment, electrostatic attraction, and chemical interactions. The foam-templated alginate network creates macropores that facilitate the diffusion of ethanol-based plant extracts. Bentonite, incorporated within the alginate matrix, exhibits negatively charged silicate layers that attract the positively charged regions of chlorophyll molecules, thereby enhancing the π-π stacking between the chlorophyll macrocycle and the clay surface (Figure 13). The gamma-irradiated chitosan coating introduces protonated amino groups (−NH_3_^+^) and hydroxyl groups (−OH), which form electrostatic and hydrogen bonds with the chlorophyll structure, particularly with its central Mg^2+^ ion and peripheral carbonyl groups. Additionally, hydrophobic interactions between the chitosan surface and the phytol tail of chlorophyll further enhance the stability of the adsorption process. In the ethanol–water medium, the association between chlorophyll and other bioactive compounds is weakened, allowing for selective removal of chlorophyll while preserving phenolic and flavonoid compounds in the extract. Consequently, the composite beads demonstrate effective chlorophyll capture and efficient preservation of plant bioactives.

**Figure 13:**
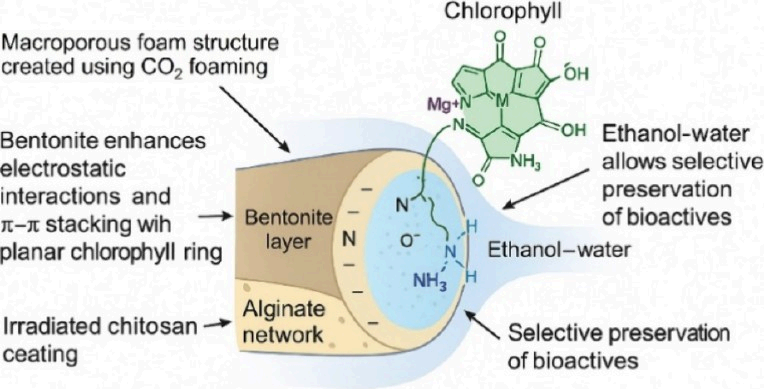
Schematic representation of the proposed chlorophyll adsorption mechanism on FP-Alg/Bent-gCS beads.

## 4 Conclusions

This study successfully developed foam-porous alginate-bentonite beads coated with gamma-irradiated chitosan (FP-Alg/Bent-gCS) as a selective and reusable adsorbent for removing chlorophyll from ethanol-based kale extracts. The CO_2_-foaming process produced a highly porous structure, while the incorporation of bentonite and the gamma-irradiated chitosan coating enhanced surface stability and charge distribution. BET analysis confirmed a high specific surface area of 75.6 m^2^·g^–1^ and a pore volume of 0.096 cm^3^·g^–1^. TEM, HRTEM, and STEM-EDS mapping of FP-Alg/Bent-gCS beads, revealing the layered morphology of bentonite platelets embedded in the alginate-chitosan matrix, as well as the homogeneous elemental distribution of Si, Al, and Ca throughout the composite. FTIR spectra indicated strong hydrogen bonding and electrostatic interactions among hydroxyl, carboxyl, and amine groups, whereas XRD patterns revealed a semicrystalline structure of chitosan integrated within the amorphous alginate-bentonite domains. Batch adsorption experiments demonstrated the rapid and efficient removal of chlorophyll. At a bead dosage of 5.0 g in 25 mL of 50% ethanol, the kale extract decreased the total chlorophyll concentration from 20.09 mg/L to 2.33 mg/L within 30 min, achieving an 88.4% removal efficiency. The adsorption kinetics followed a pseudo-second order (PSO) model, indicating that the process was dominated by chemisorption due to electrostatic attraction between the positively charged -NH_3_^+^ groups of gamma-irradiated chitosan and the negatively charged chlorophyll molecules. Importantly, colorimetric total phenolic content (TPC) and total flavonoid content (TFC) assays showed no significant change in color intensity before and after treatment, confirming that hydrophilic phenolics and flavonoids were preserved. This demonstrates that FP-Alg/Bent-gCS beads act as a selective adsorption gate, binding hydrophobic chlorophyll molecules while retaining beneficial plant bioactives. Reusability testing over seven cycles revealed that the beads maintained over 85% removal efficiency for the first five cycles, with a minor decline in performance after the sixth cycle due to partial surface deformation and active site saturation. EDS and XRD analyses after reuse confirmed that the elemental composition (Si, Al, Ca) and structural stability were largely retained. In conclusion, the FP-Alg/Bent-gCS beads exhibit excellent porosity, selectivity, and durability, providing an eco-friendly and low-cost approach for the purification of pigments from plant extracts. This material holds strong potential for applications in green extraction and purification processes in the food, herbal, and cosmetic industries.

## Supplementary Material



## Data Availability

The data supporting this study are available within the manuscript.

## Notes

Pseudo-first-order kinetic plots, additional adsorption fitting parameters (Table S1), and supplementary characterization data (Figure S1) (PDF)
