# Supplementary material for: Foam-Porous Alginate-Bentonite Beads Coated with Gamma-Irradiated Chitosan for Selective Chlorophyll Removal with Preservation of Plant Bioactives
Source: ACS Omega. 2026 Mar 4;11(10):16300–10. doi: 10.1021/acsomega.5c11997 (PMC13000570; doi:10.1021/acsomega.5c11997)
Supplement: Supplementary file 1 [file ao5c11997_si_001.pdf]

Foam-Porous Alginate-Bentonite Beads Coated with Gamma-Irradiated Chitosan for Selective Chlorophyll Removal with Preservation of Plant Bioactives

Titiya Meechai[1], Pitchapa Pittayavinai[1], Narudom Srisawang[1], Jintapat Nateewattana[2], Tanutta Amnuaywattanakul[2] and Phitchan Sricharoen[2*]

[1]Faculty of Dentistry, Bangkokthonburi University, Thawi Watthana, Bangkok 10170, Thailand.

[2]Division of Health, Cosmetic and Anti-Aging Technology, Faculty of Science and Technology, Rajamangala University of Technology Phra Nakhon, Bangkok 10800, Thailand.

*Corresponding author; e-mail: phitchan.s@rmutp.ac.th (P. Sricharoen)

The Pseudo-First-Order (PFO) model

The pseudo-first-order (PFO) kinetic model was evaluated according to the Lagergren equation:

$$\ln(q_e - q_t) = \ln(q_e) - k_1\, t$$

where

q_e= adsorption capacity at equilibrium (mg g⁻¹)

q_t= adsorption capacity at time t (mg g⁻¹)

k_1= PFO rate constant (min⁻¹)

t = contact time (min)

Table S1. Pseudo-first-order (PFO) kinetic parameters for chlorophyll adsorption

Bead dosage	q_e (mg g^{-1})	k_1 (min^{-1})	R^2 (PFO)
3.0 g	0.14	0.038	0.91
5.0 g	0.10	0.031	0.89

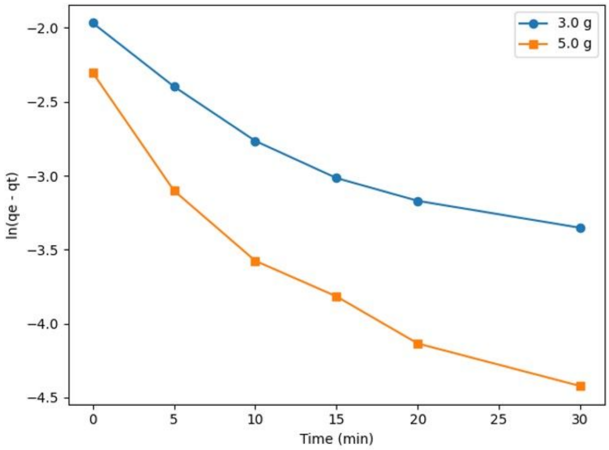

Figure S1. Linear pseudo-first-order (PFO) kinetic plots (ln(q_e - q_t) versus time) for chlorophyll adsorption onto FP-Alg/Bent-gCS beads at different bead dosages (3.0 g and 5.0 g) in 50% ethanol.
